# Diagnosis of Hemophagocytic Lymphohistiocytosis in Pyrexia of Unknown Origin: A Case Report

**DOI:** 10.31729/jnma.8626

**Published:** 2024-06-30

**Authors:** Khusbu Thapa, Bikranta Bikram Kharel, Shreya Shrestha, Tanbir Ikram

**Affiliations:** 1 Department of Hematology, Blood and Multispeciality Hospital and Research Center Private Limited, Jawalakhel, Lalitpur, Nepal; 2 Department of Emergency Medicine, Lifeline Hospital, Jhapa, Nepal; 3 Nepal Medical College and Teaching Hospital, Attarkhel, Jorpati, Nepal

**Keywords:** *case report*, *hemophagocytic lymphohistiocytosis*, *Nepal*, *PET scan*, *pyrexia*

## Abstract

A case of a 61-year male presented with chief complaints of fever for three months. Diagnosis of hemophagocytic lymphohistiocytosis was made after liver biopsy when Positron-Emission Tomography revealed multiple fluoro deoxy glucose avid lesions in the liver. The patient of this disease typically presents with fever, splenomegaly, hyperferritinemia, hypertriglyceridemia, raised liver enzymes, and marrow features suggestive of hemophagocytosis. The treatment is usually systemic corticosteroids, chemotherapy with etoposide, cyclosporine, or hematopoietic stem cell transplantation, depending on the cause. A diagnosis like hemophagocytic lymphohistiocytosis can easily be missed in developing countries with low resources like Nepal. Hence, this disease should be suspected in patients presenting with pyrexia of unknown origin by treating physicians.

## INTRODUCTION

Hemophagocytic Lymphohistiocytosis (HLH) is a rare, fatal condition due to unregulated and uncontrolled hyperinflammation initially reported more in children. However, the cases have increased in adults as well.^1,2^ Primary or Hereditary HLH is caused by a mutation in genes (the most common one being the PRF1 gene, others are UNC13D, STX11, and STXBP2), seen mostly in infants. Secondary or Acquired HLH is more commonly seen in adults and could be triggered by infection, autoimmune disease, or malignancy.^1,2,7^ The diagnosis of HLH has been a challenge due to its diverse clinical presentation and multiple mimics. Most reported clinical presentations include fever, splenomegaly, and laboratory reports show bicytopenia, hyperferritinemia, hypofibrinogenemia, hypertriglyceridemia, and biopsy showing hemophagocytosis in reticuloendothelial organs like the liver, spleen, or lymph node.^3,4^

In 2004, revised diagnostic criteria were introduced by the Histiocyte Society which is most commonly used for diagnosis. Three new criteria were added to the 1991 diagnostic criteria.^1,5^

A molecular diagnosis consistent with HLH or five of the following eight criteria (Table 1).

**Table 1:** HLH-2004 diagnostic criteria.

**A. Initial Diagnostic Criteria**	
1.	Fever
2.	Splenomegaly
3.	Cytopenia (minimum 2 cell lines reduced from the following: Hemoglobin <90 g/L, Neutrophil <100 × 109 /L, Platelets <1 × 109 /L)
4.	Hyperferritinemia > 500 mg/L
5.	Hypofibrinogenemia or <1.5 g/L Hypertriglyceridemia >3 mmol/L
**B. New Diagnostic Criteria**	
6.	High-soluble CD25 >2400 U/mL
7.	Hemophagocytosis in lymph node, bone marrow
8.	Low or absent NK cell activity

The HLH treatment protocol (HLH-2004) consisted of 8 weeks of initial treatment followed by a continuation phase, both phases including etoposide and dexamethasone.^6^ However, the mortality rate remains high in HLH despite appropriate treatment.^7^

## CASE REPORT

A 61-year-old male presented to the hospital with chief complaints of fever for 3 months associated with generalised body weakness. In previous health centres, initial workup for fever was done but a definitive diagnosis was not made. On initial assessment, his vitals showed T-103F, HR-124b/m, BP-130/70 mm Hg, RR-14 breaths/min.

Investigations revealed bicytopenia (hemoglobin- 8.0gm/dl, Platelets-66000/cu. mm), Tropical panel (Scrub typhus, Dengue serology, Widal test, Chikungunya, RK39, blood culture, serology), RT-PCR for Epstein Barr Virus were negative. Bone marrow aspiration showed normocellular marrow fragments with an increase in megakaryopoiesis and was negative for malignancy. Lactate Dehydrogenase (1503U/l) and Ferritin (>2000mg/ml) were high. Ultrasonography (Abdomen + Pelvis) (USG) suggested hepatomegaly and Computed Tomography(Abdomen and Pelvis) revealed no significant lymph nodes. Further investigations revealed Triglyceride (TG)level- 270 mg/dl (<200 mg/dl) and Fibrinogen- 2.0 gm/dl (1.54 gm/dl).

**Figure 1:**
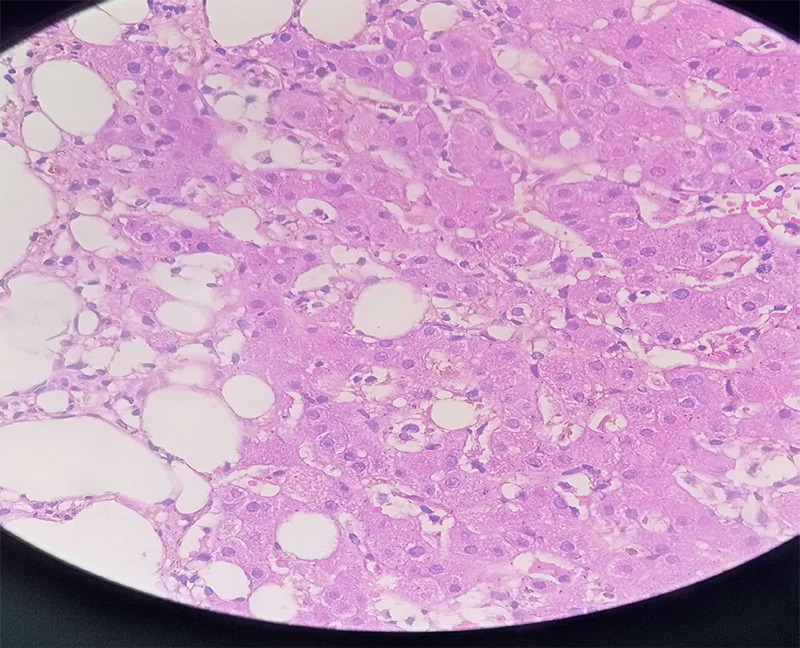
Histopathological photograph showing histiocytes (phagocytosed leukocytes and erythrocytes)

Antinuclear Antibody was positive and PET scan of the whole body revealed hepatosplenomegaly and FDG avid lesions in the liver, which pointed towards secondary metastases in the liver. However, tumor markers like Carcinoembryonic antigen (CEA), Cancer Antigen 19-9 (CA 19-9) and Alfa-fetoprotein (AFP) were negative. Liver biopsy was later done which showed diffuse infiltration in the hepatic sinusoids by histiocytes demonstrating phagocytosed leukocytes and erythrocytes with no features of malignancy. (Figure 1). With the above findings, a definitive diagnosis of HLH was made.

Treatment was started with Inj Dexamethasone (10mg/m^2^) and Etoposide. The patient improved and he had no single episode of fever after steroid administration. The patient was discharged after 5 days on Inj Dexamethasone daily for a total of 14 days and Inj Etoposide 150 mg twice weekly.

## DISCUSSION

Hemophagocytic Lymphohistiocytosis is a rare disease and there are very few cases of HLH reported in Nepal. It has multifactorial pathogenesis due to immune hyperactivation, which is triggered either by underlying inflammation, infection, or neoplasm.^4^ It is similar to other inflammatory disorders and has complex diagnostic criteria due to which prompt and accurate diagnosis is difficult. Without active intervention, the progression of the disease is concerning and is associated with high mortality.^8^

It is important to work up for underlying causes because in both familial and sporadic cases immune hyperactivity is commonly triggered by underlying infections.^9^ The present case presented with unexplained fever for 3 months. In our case, all the infective causes were ruled out and ANA came positive and autoimmune association was assumed.

In HLH evidence of phagocytosis of hematopoietic cells can be seen in the reticuloendothelial system.^10^ In the presenting case, Lab parameters pointed towards HLH and Imaging revealed lesions in the liver. A liver biopsy showed diffuse infiltration in the hepatic sinusoids by histiocytes demonstrating phagocytosed leukocytes and erythrocytes without features of malignancy. Management differs according to etiology. HLH due to immune dysregulation such as those associated with macrophage activation syndrome are treated with immunosuppressants whereas those with underlying mutations require bone marrow transplantation and hence are initially treated with chemotherapy.^4^

Our case, after diagnosis, was managed with IV steroids and Etoposide which drastically improved the patient's symptoms along with the subsidence of fever.

Diagnosis and management of HLH requires a multispecialty approach from and not limited to Internal Medicine, Diagnostic Radiology, Interventional Radiology, Pathology, Hematologist, Microbiology, Immunology, Rheumatology. In low resource countries like Nepal, there are only few centres with multispecialty services and lack of knowledge regarding the disease, its prevalence and awareness further delays prompt diagnosis and planning of management.
